# The role of human melanoma cell ICAM-1 expression on lymphokine activated killer cell-mediated lysis, and the effect of retinoic acid

**DOI:** 10.1038/sj.bjc.6690551

**Published:** 1999-07

**Authors:** C L Alexander, M Edward, R M MacKie

**Affiliations:** Department of Dermatology, The Robertson Building, University of Glasgow, Glasgow G12 8QQ, UK

**Keywords:** melanoma, ICAM-1, LAK cells, retinoic acid

## Abstract

Intercellular adhesion molecule (ICAM-1) exists as a membrane-associated form (mICAM-1) on the surface of tumour cells as well as a soluble form (sICAM-1). This study analyses the ability of all- *trans* retinoic acid (RA) to alter both sICAM and mICAM-1 expression in C8161 and Hs294T human melanoma cell lines and investigates the involvement of ICAM-1 in the interaction between tumour and lymphokine-activated killer (LAK) cells using the Cr-51 release assay. Our data showed that 4-day pretreatment of the tumour cells with 10^−7^ M RA and 10^−6^ M RA induced an increase in lysis of both cell lines and also increased mICAM-1 expression without having any effect on sICAM-1 levels. Addition of blocking ICAM-1 antibody (10 μg ml^−1^) to the C8161 cells at an effector:tumour cell ratio of 40:1 caused a 2.3-fold reduction in lysis of tumour cells and a 3-fold reduction in lysis of RA-treated cells. Blocking ICAM-1 antibody at optimum concentrations of 5 μg ml^−1^ reduced lysis 1.8-fold in control Hs294T cells and 1.3-fold in RA-treated cells. Blocking the HLA–ABC complex had no effect on lysis. The more highly metastatic C8161 cells were found to secrete 4-fold greater levels of sICAM-1 than the poorly metastatic Hs294T cells and addition of sICAM-1 to the assay failed to affect lysis of either cell line but did induce a 2-fold decrease in lysis of RA-treated C8161 cells. Collectively, these data provide further evidence for ICAM-1 involvement in the tumour/LAK cell response and indicates that the RA-induced increase in mICAM-1 levels are partly responsible for the increase in susceptibility of the tumour cells. sICAM-1 appears to be unimportant in evasion of the tumour cells from LAK cell lysis, but may play a role in evasion of RA-treated C8161 cells. © 1999 Cancer Research Campaign


*British Journal of Cancer* (1999) **80**(10), 1494–1500
© 1999 Cancer Research Campaign
Article no. bjoc.1999.0551

### The role of human melanoma cell ICAM-1 expression on lymphokine activated killer cell-mediated lysis, and the effect of retinoic acid

**CL Alexander, M Edward and RM MacKie**

Department of Dermatology, The Robertson Building, University of Glasgow, Glasgow G12 8QQ, UK

**Summary** Intercellular adhesion molecule (ICAM-1) exists as a membrane-associated form (mICAM-1) on the surface of tumour cells as well as a soluble form (sICAM-1). This study analyses the ability of all-*trans* retinoic acid (RA) to alter both sICAM and mICAM-1 expression in C8161 and Hs294T human melanoma cell lines and investigates the involvement of ICAM-1 in the interaction between tumour and lymphokine-activated killer (LAK) cells using the Cr-51 release assay. Our data showed that 4-day pretreatment of the tumour cells with $10^{-7}$ M RA and $10^{-6}$ M RA induced an increase in lysis of both cell lines and also increased mICAM-1 expression without having any effect on sICAM-1 levels. Addition of blocking ICAM-1 antibody (10 μg $ml^{-1}$) to the C8161 cells at an effector:tumour cell ratio of 40:1 caused a 2.3-fold reduction in lysis of tumour cells and a 3-fold reduction in lysis of RA-treated cells. Blocking ICAM-1 antibody at optimum concentrations of 5 μg $ml^{-1}$ reduced lysis 1.8-fold in control Hs294T cells and 1.3-fold in RA-treated cells. Blocking the HLA–ABC complex had no effect on lysis. The more highly metastatic C8161 cells were found to secrete 4-fold greater levels of sICAM-1 than the poorly metastatic Hs294T cells and addition of sICAM-1 to the assay failed to affect lysis of either cell line but did induce a 2-fold decrease in lysis of RA-treated C8161 cells. Collectively, these data provide further evidence for ICAM-1 involvement in the tumour/LAK cell response and indicates that the RA-induced increase in mICAM-1 levels are partly responsible for the increase in susceptibility of the tumour cells. sICAM-1 appears to be unimportant in evasion of the tumour cells from LAK cell lysis, but may play a role in evasion of RA-treated C8161 cells.

**Keywords:** melanoma; ICAM-1; LAK cells; retinoic acid

Intercellular adhesion molecule-1 (ICAM-1, CD54), a member of the immunoglobulin superfamily (Staunton et al, 1988), acts as a counter-receptor for leucocyte function associated antigen-1 (LFA-1, CD11a/CD18) (Marlin and Springer, 1987) present on the surface of T-lymphocytes, lymphokine-activated killer (LAK) cells and natural killer cells. Melanoma cells are among the various cell types known to express membrane ICAM-1 (mICAM-1), which may be crucial for the interaction with the immune system. mICAM-1 levels are induced by cytokines such as interferon γ (IFN-γ), tumour necrosis factor α (TNF-α) and interleukin-1 (IL-1) (Rothlein et al, 1988) which are reported to make the tumour cells more susceptible to lysis by cells of the immune system (Webb et al, 1991). One possible mechanism as to how tumour cells evade immunosurveillance by lymphocytes is through the release of a soluble form of ICAM-1 (sICAM-1) which may exist as a multimeric form or complexed with other proteins, and is of lower molecular mass (80 kDa) (Rothlein et al, 1991) than membrane ICAM-1 (90 kDa) (Marlin and Springer, 1987). Serum levels have been reported to increase in various disease states (Banks et al, 1993) which include human malignant melanoma (Harning et al, 1991); however, the biological consequences of such increases remains controversial. It is possible that binding of sICAM-1 to the LFA-1 receptor could prevent the lymphocyte from interacting with mICAM-1 and thus prevent tumour cell lysis occurring. This is supported by reports from various groups (Becker et al, 1992; Altomonte et al, 1993) but which is in contrast to the recent data from Young et al (1997) who reported on the inability of sICAM-1 to affect tumour cell lysis.

The biologically active derivative of vitamin A, retinoic acid (RA), is known to have effects on tumour growth, differentiation, adhesive properties and apoptosis. It has been used in cancer therapy and is thought to render many tumour cells more susceptible to attack by effector cells of the immune system (Smith et al, 1992). The retinoids exert their physiological actions by binding to nuclear receptors which dimerize and interact with specific sites on the DNA termed the RA response elements (RARE). The promoter region of the gene for ICAM-1 has been shown to contain a RARE (Aoudjit et al, 1994), suggesting RA has the ability to alter ICAM-1 expression (Cilenti et al, 1995). RA is known to increase ICAM-1 expression in several cell types including melanoma cells (Bouillon et al, 1991), mast cells (Babina et al, 1997) and epithelial cells (Gao and Mackenzie, 1996). In this study, we investigate the effect of RA on its ability to alter both membrane and soluble ICAM-1 expression and the immunological consequences of such treatments in vitro. We compare the highly metastatic C8161 human melanoma cell line with the low metastatic Hs294T cell line, focusing on modulation of the interaction between untreated/treated tumour cells and LAK cells.

*Received 9 March 1998*
*Revised 26 January 1999*
*Accepted 27 January 1999*

*Correspondence to:* CL Alexander

### MATERIALS AND METHODS

### Materials

Eagle's minimal essential medium (EMEM), Dulbecco's modified Eagle medium (DMEM), RPMI-1640, phosphate-buffered saline (PBS), fetal calf serum (FCS), penicillin–streptomycin, L-glutamine, trypsin and Nunclon tissue culture plastics were obtained from Life Technologies Ltd (Paisley, UK). All-*trans* RA was purchased from Sigma Chemical Co (Dorset, UK) while Ficoll-hypaque research grade was from Pharmacia Biotech, (Hertfordshire, UK). Chromium-51 was obtained from Amersham International plc (Buckinghamshire, UK). Human recombinant interleukin-2 (h-rIL2), recombinant sICAM-1, fluorescein isothiocyanate (FITC)-conjugated anti-human ICAM-1 antibody and the monoclonal mouse anti-human ICAM-1 blocking antibody were from R&D Systems Europe (Abingdon, UK). The non-specific mouse IgG control antibody was from the Scottish Antibody Production Unit (SAPU, Law Hospital, UK). Mouse anti-human HLA–ABC antibody was purchased from Serotec Ltd (Oxford, UK).

### Cell culture

The highly metastatic human melanoma C8161 cell line was established from an abdominal wall metastasis and characterized by Welch et al (1991) and was kindly supplied by Mary JC Hendrix (University of Arizona, Tucson, AZ, USA). The low metastatic Hs294T cell line (Creasey et al, 1979), derived from a lung node metastasis, was obtained from the American Tissue Culture Collection (ATCC, HTB-140). Both cell lines were routinely cultured in EMEM supplemented with 10% heat-inactivated FCS, penicillin (100 units $ml^{-1}$) and streptomycin (100 μg $ml^{-1}$). Cells were maintained in a humidified atmosphere of 5% carbon dioxide in air at 37°C.

### Methods

### *Preparation of lymphocytes*

Eighty millilitres of heparinized blood from a healthy volunteer were subjected to Ficoll density gradient centrifugation and the peripheral blood mononuclear cell layer removed. Adherent cells were removed by two cycles of adherence to tissue culture plastic. The non-adherent peripheral blood lymphocytes (PBL) were collected and maintained in RPMI supplemented with 10% FCS and lymphocytes activated by the addition of 2 nM h-rIL2 to $10^6$ cells for at least 6 days to produce LAK cells.

### Assessment of PBL and LAK cell-mediated tumour cell lysis

### *Chromium-51 release assay*

A total of $5 \times 10^3$ tumour cells, either untreated or treated with $10^{-10}$ M to $10^{-6}$ M RA for 4 days, were radiolabelled with Chromium-51 (400 μCi $10^{-7}$ cells, specific activity 200–500 mCi $mg^{-1}$) and incubated for 2 h at 37°C. Excess radiolabelled chromium was removed and labelled tumour cells incubated with varying numbers of lymphocytes to produce the effector:tumour cell ratio (E:T). After 4-h incubation, a 70 μl sample of supernatant was removed for liquid scintillation counting (Canberra-Packard). Spontaneous release was measured by incubating the tumour cells in complete media and maximum release determined by lysing the cells in 0.1 M hydrochloric acid. For all valid assays, spontaneous release from both treated and untreated tumour cells was always less than 10% of the maximum release. Blocking anti-ICAM-1 antibody was used at concentrations ranging from 1 μg $ml^{-1}$ to 25 μg $ml^{-1}$, and a non-specific mouse IgG antibody used as an isotype control. A melanoma-specific antibody (NK1/C3; TCS Biologicals) which recognizes both intracellular and cell-surface epitopes was also used as a control antibody, and had the same effect as the isotype control. Both antibodies were incubated for 1 h, 37°C with the tumour cells before the addition of the LAK cells. sICAM-1 was added over a range of concentrations (25 ng $ml^{-1}$ to 125 ng $ml^{-1}$) and was present throughout the lysis assay.

### *Quantitative analysis of cytotoxicity*

The percentage of lysis occurring was calculated as follows: % specific lysis = (dpm experimental well – dpm spontaneous release) ÷ (dpm maximal release – dpm spontaneous release).

### Immunofluorescent analysis of mICAM-1

### *Flow cytometry*

Tumour cells were prepared for fluorescent-activated cell sorter analysis by the addition of 10 μl of FITC-conjugated ICAM-1 antibody (equivalent to one test as described by R&D systems) to $10^6$ tumour cells and incubated for 15 min. Excess antibody was removed by washing the cells twice in PBS, and fluorescent staining analysed using a FACscan (Becton Dickinson). Intensity of fluorescence was recorded on a logarithmic scale, and corresponding background fluorescence intensity obtained using tumour cells minus antibody was always less than 0.6 units.

### Soluble ICAM-1 assay

The procedure for measuring sICAM-1 was as described in the R&D Systems parameter handbook. Briefly, 100 μl of anti-ICAM-1 horseradish peroxidase conjugate was added to each well which had been coated with a murine antibody to human ICAM-1. This was followed by the addition of diluted supernatant from cells pretreated with RA ($10^{-10}$ M to $10^{-6}$ M) for 7 days. The plate was incubated for 2 h at room temperature before the contents of each well were removed. Wells were washed 6 times with 300 μl of wash buffer, after which 100 μl of substrate was added to each well and left to incubate at room temperature for 30 min. One hundred microlitres of the stop solution was added to each well and the optical density of the plate read in a microtitre plate reader set at 450 nm with correction of 620 nm.

### Statistical analysis of data

Data were analysed statistically using the two-tailed Student's *t*-test and values expressed as the average ± s.e.m. of ten separate samples from a single experiment with the exception of the sICAM-1 lysis experiments which were performed in triplicate.

### RESULTS

### Cytotoxicity assay

Unactivated PBL failed to induce lysis of both untreated/treated C8161 cells and Hs294T cells at all E:T ratios tested (data not shown). Pretreatment of the C8161 cells for 4 days using the lower concentrations of RA ($10^{-10}$ M to $10^{-8}$ M) had no significant effect on LAK cell-mediated tumour cell lysis (Figure 1). Nevertheless, pretreatment with $10^{-7}$ M RA increased lysis of the C8161 cells 1.8-fold ($P < 0.05$) at the 20:1 ratio, and 1.4-fold ($P < 0.05$) at the 40:1 ratio (Figure 1). Pretreatment of the C8161 cells with $10^{-6}$ M RA induced a 2-fold increase in lysis at the 20:1 ratio ($P < 0.05$) (Figure 1) with similar increases of 1.7-fold ($P < 0.05$) and

© 1999 Cancer Research Campaign

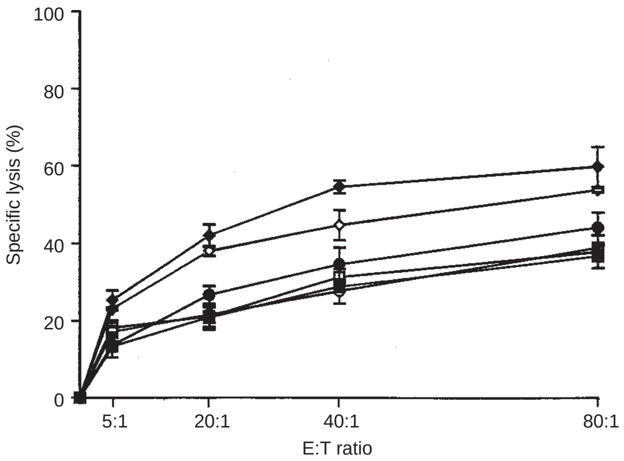

**Figure 1** IL-2-activated PBL lysis of C8161 cells (□) and C8161 cells pretreated with $10^{-10}$ M RA (■); $10^{-9}$ M RA (○); $10^{-8}$ M RA (●); $10^{-7}$ M RA (◇) and $10^{-6}$ M RA (◆)

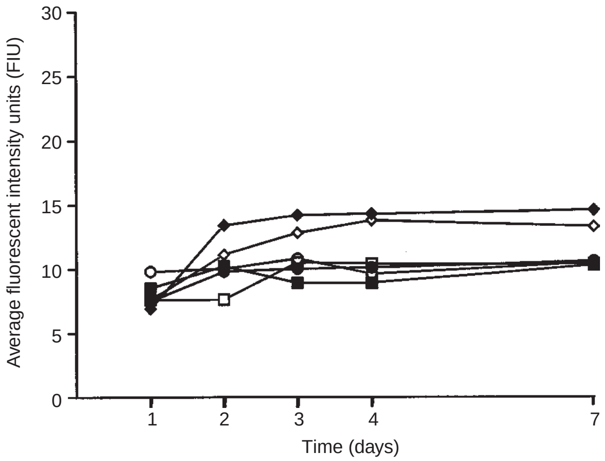

**Figure 3** Flow cytometric analysis of mICAM-1 expression in C8161 cells (□) and C8161 cells pretreated with $10^{-10}$ M RA (■); $10^{-9}$ M RA (○); $10^{-8}$ M RA (●); $10^{-7}$ M RA (◇) and $10^{-6}$ M RA (◆)

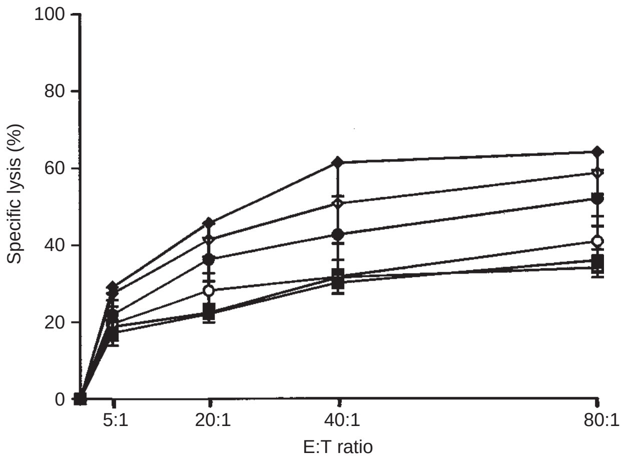

**Figure 2** IL-2-activated PBL lysis of Hs294T cells (□) and Hs294T cells pretreated with $10^{-10}$ M RA (■); $10^{-9}$ M RA (○); $10^{-8}$ M RA (●); $10^{-7}$ M RA (◇) and $10^{-6}$ M RA (◆)

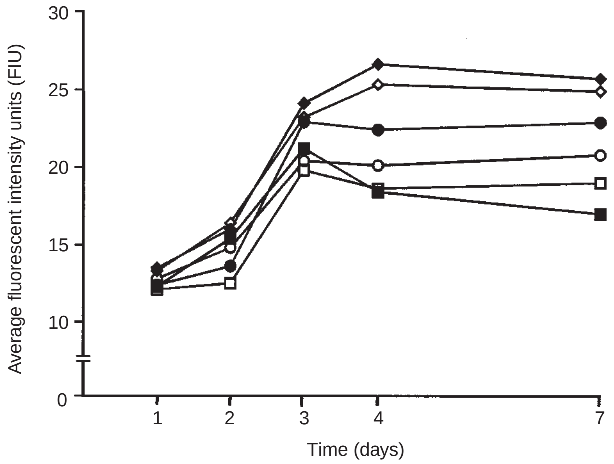

**Figure 4** Flow cytometric analysis of mICAM-1 expression in Hs294T cells (□) and C8161 cells pretreated with $10^{-10}$ M RA (■); $10^{-9}$ M RA (○); $10^{-8}$ M RA (●); $10^{-7}$ M RA (◇) and $10^{-6}$ M RA (◆)

1.6-fold ($P < 0.05$) at the 40:1 and 80:1 ratios respectively (Figure 1).

Using the Hs294T cells, 4 days pretreatment with $10^{-10}$ M to $10^{-8}$ M RA failed to significantly alter tumour cell lysis (Figure 2). In contrast, pretreatment of the Hs294T cells with $10^{-7}$ M and $10^{-6}$ M RA significantly increased lysis 1.6-fold ($P < 0.05$) and 2-fold ($P < 0.05$) respectively at the 20:1 E:T ratio (Figure 2). Similar values were obtained at the 40:1 ratio where lysis was increased 1.4-fold ($P < 0.05$) and 2-fold ($P < 0.005$) using $10^{-7}$ M and $10^{-6}$ RA respectively (Figure 2).

Pretreatment of the tumour cells for 4 days with $10^{-5}$ M RA was also examined but viability measurements (using trypan blue staining) revealed a significant decrease in viability of both cell lines using this concentration of drug. Therefore, for all further lysis experiments, $10^{-6}$ M RA was chosen as this induced the optimum increase in lysis whilst maintaining cell viability.

### Flow cytometric analysis

Fluorescent intensity levels representing mICAM-1 expression over a 7-day period in control C8161 cells ranged from, on average, 7.6 to 10.4 (Figure 3). Exposure of the C8161 cells over a 7-day period with RA ($10^{-10}$ M to $10^{-8}$ M) did not significantly alter mICAM-1 expression (with values of average fluorescent intensity ranging from 8.5 to 10.6). After 1 day treatment of the C8161 cells with $10^{-7}$ M and $10^{-6}$ M RA, mICAM-1 expression was not altered but levels began to increase 1.4-fold using $10^{-7}$ M RA and 1.7-fold using $10^{-6}$ M RA by day 2 (Figure 3). This increase was maintained with 4 days treatment, where $10^{-7}$ M RA induced a 1.3-fold increase in mICAM-1 expression and a 1.4-fold increase using $10^{-6}$ M RA (Figure 3).

Expression of mICAM-1 in control Hs294T cells was on average 2-fold higher than in the C8161 cells. Pretreatment of the Hs294T cells over a 7-day period with $10^{-10}$ M to $10^{-9}$ M RA had

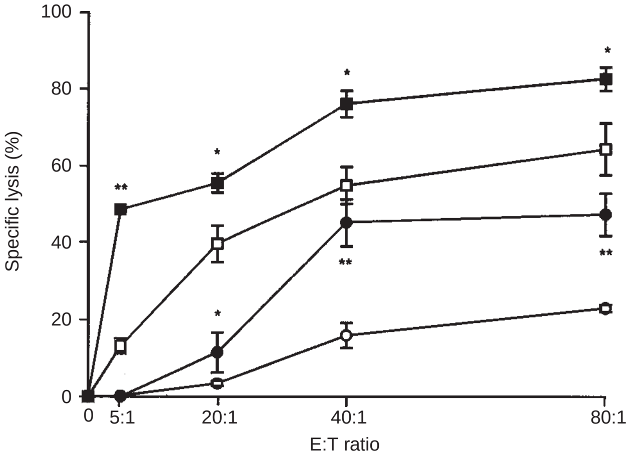

**Figure 5** IL-2-activated PBL lysis of Hs294T cells pretreated without (□) and with (■) $10^{-6}$ M RA and lysis of C8161 cells pretreated without (○) and with (●) $10^{-6}$ M RA. Values are represented as the average ± s.e.m. where *$P < 0.05$ and **$P < 0.005$

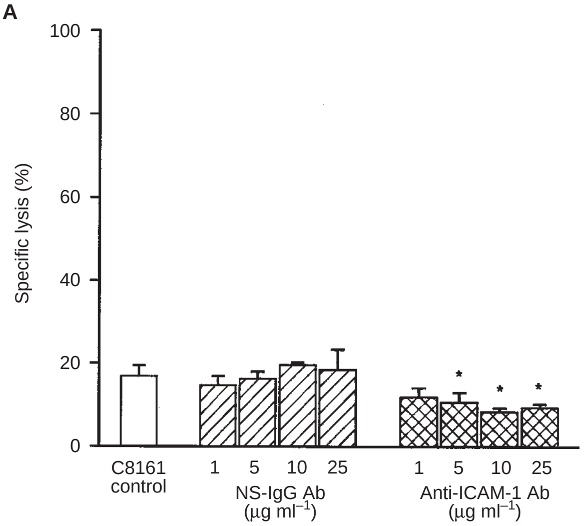

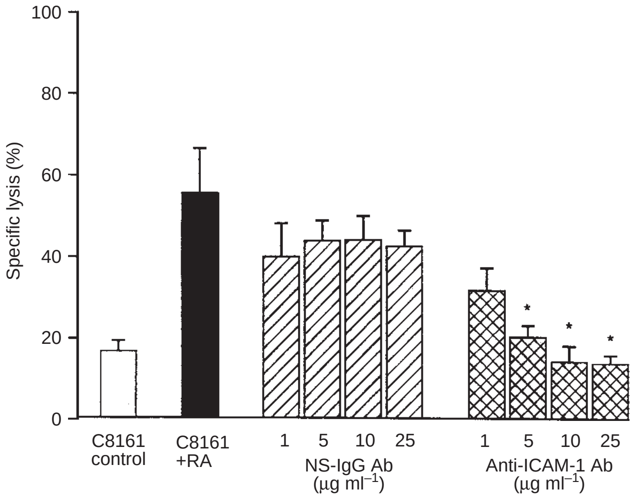

**Figure 6** (**A**) Effect of blocking anti-ICAM-1 antibody (cross-hatched bar) on the lysis of C8161 cells (open bar) at an E:T ratio of 40:1. Non-specific IgG antibody was also included as a control (hatched bar). Values are represented as the average ± s.e.m. where *$P < 0.0005$ and represents comparison of control IgG with anti-ICAM-1 antibody. (**B**) Effect of blocking anti-ICAM-1 antibody (cross-hatched bar) on the lysis of C8161 cells pretreated for 4 days with $10^{-6}$ M RA (solid bar) at an E:T ratio of 40:1. Non-specific IgG antibody was also included as a control (hatched bar). Values are represented as the average ± s.e.m. where *$P < 0.005$ and represents comparison of control IgG with anti-ICAM-1 antibody no effect on mICAM-1 expression. However, using $10^{-8}$ M RA, ICAM-1 expression was marginally increased from 19 to 23 fluorescent intensity units on day 3, which was similar by day 4 and day 7 (Figure 4). After 2 days treatment with $10^{-7}$ M RA and $10^{-6}$ M RA, mICAM-1 expression was increased by both treatments 1.3-fold, remaining at this level by 4 days with $10^{-7}$ M RA pretreatment and increasing 1.4-fold with $10^{-6}$ M RA (Figure 4).

### Addition of blocking ICAM-1 antibody to the cytotoxicity assay

Due to the variation in LAK cell populations between individuals, a lysis assay was set up using the same population of lymphocytes as used in the blocking assays, to examine the effect of 4 days pretreatment with $10^{-6}$ M RA on lysis of the tumour cells. Then, $10^{-6}$ M RA-treated C8161 and Hs294T cells incubated with LAK cells for a period of 4 h were found to become more susceptible to lysis than untreated control cells. In the C8161 cell line (Figure 5), lysis was increased 3.3-fold from 3.5% to 11.5% at the 20:1 ratio ($P < 0.05$) with similar increases of 2.8-fold ($P < 0.005$) and 2.1-fold ($P < 0.005$) at the 40:1 and 80:1 ratios respectively. A similar pattern was observed using the Hs294T cell line (Figure 5) where lysis was increased 3.7-fold at the 5:1 ratio from 13.1% to 48.4% ($P < 0.005$), from 39.5% to 55.5% at the 20:1 ratio (1.4-fold) and from 54.9% to 76.1% at the 40:1 ratio (1.4-fold) with *P*-values < 0.05.

Blocking ICAM-1 antibody was added to the cytotoxicity assay at the 40:1 ratio to untreated tumour cells and to tumour cells that had been exposed to $10^{-6}$ M RA for 4 days. For all comparisons, lysis on addition of blocking ICAM-1 antibody was compared to a non-specific IgG control antibody, while use of a melanoma cell-specific control antibody had no effect. A control of untreated tumour cells was run with each experiment to show the increase in lysis induced by RA was consistent with each experiment despite the slight differences in lysis of the tumour cells using different populations of LAK cells. Addition of 5 μg $ml^{-1}$ of anti-ICAM antibody at the 40:1 ratio (Figure 6A) caused a highly significant 1.5-fold decrease in lysis of control C8161 cells (from 16.2% to 10.7%, $P < 0.0005$). Optimum inhibition was achieved using 10 μg $ml^{-1}$ of anti-ICAM-1 antibody which reduced lysis from 19.6% to 8.4% (a 2.3-fold difference, $P < 0.0005$). This inhibition was maintained at the higher concentration of antibody.

A similar pattern was also observed in RA-treated cells (Figure 6B) where lysis was reduced at 5 μg $ml^{-1}$ anti-ICAM-1 antibody from 43.7% to 20.5% ($P < 0.005$). Optimum inhibition was also achieved using 10 μg $ml^{-1}$ anti-ICAM antibody which induced a threefold reduction in lysis of the cells from 44.0% to 14.4% ($P < 0.005$), which was similar at the higher concentration of antibody.

In the Hs294T cell line (Figure 7A), it was found that at the 40:1 ratio, blocking anti-ICAM-1 antibody at the optimal concentration

© 1999 Cancer Research Campaign

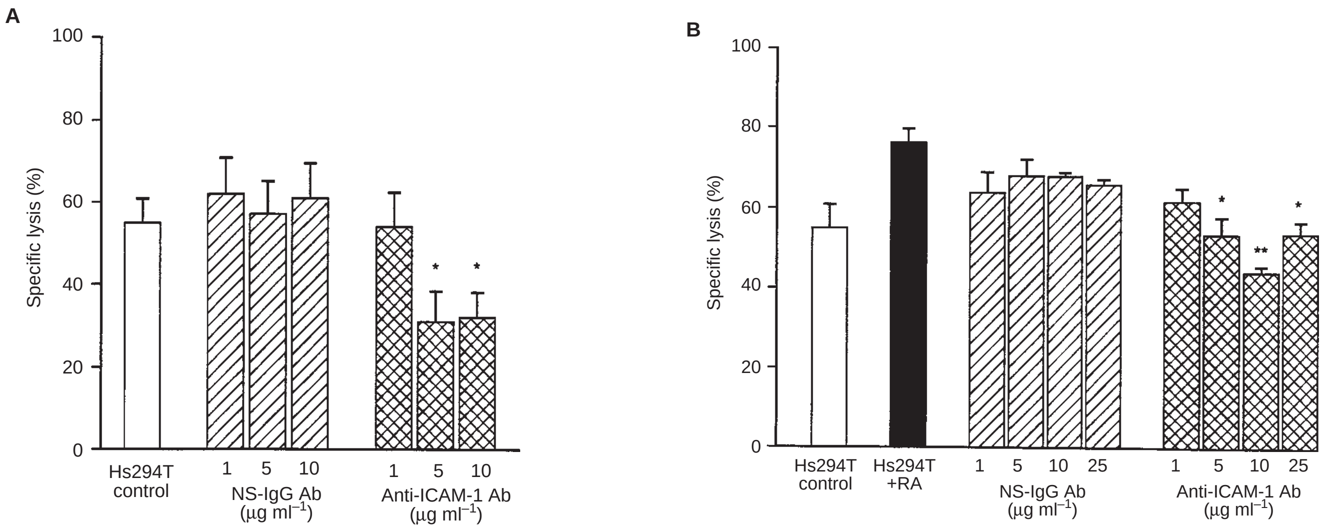

**Figure 7** (**A**) Effect of blocking anti-ICAM-1 antibody (cross-hatched bar) on the lysis of Hs294T cells (open bar) at an E:T ratio of 40:1. Non-specific IgG antibody was also included as a control (hatched bar). Values are represented as the average ± s.e.m. where *$P < 0.001$ and represents comparison of control IgG with anti ICAM-1 antibody. (**B**) Effect of blocking anti-ICAM-1 antibody (cross-hatched bar) on the lysis of Hs294T cells pretreated for 4 days with $10^{-6}$ M RA (solid bar) at an E:T ratio of 40:1. Non-specific IgG antibody was also included as a control (hatched bar). Values are represented as the average ± s.e.m. where *$P < 0.05$ and represents comparison of control IgG with anti-ICAM-1 antibody

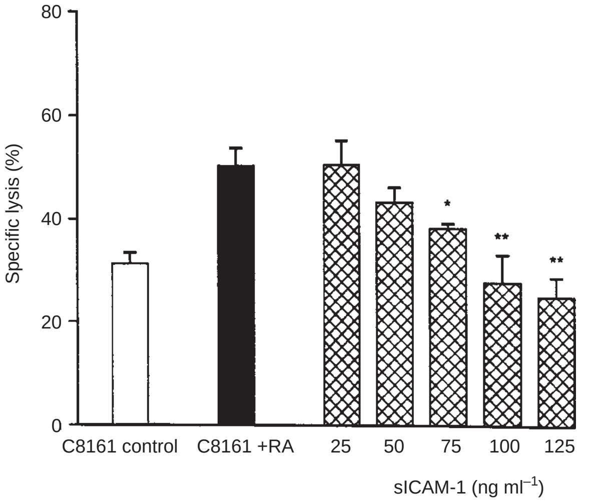

**Figure 8** Effect of soluble ICAM-1 (cross-hatched bars) on the lysis of C8161 cells pretreated with $10^{-6}$ M RA (solid bar)

of 5 µg ml$^{-1}$ significantly reduced lysis of control cells 1.8-fold from 57.1% to 31.1% ($P < 0.001$). This reduction was also observed using 10 µg ml$^{-1}$ anti-ICAM-1 antibody that reduced lysis 1.9-fold ($P < 0.001$). On treatment of Hs294T cells with $10^{-6}$ M RA (Figure 7B) lysis was significantly reduced using anti-ICAM-1 antibody at 5 µg ml$^{-1}$ which caused a 1.3-fold reduction in lysis from 67.8% to 53.4% ($P < 0.05$). Similar reductions were observed at all other antibody concentrations tested.

### ELISA assay for measuring sICAM-1 expression

C8161 cells secreted basal of 43.6 fg ICAM-1 per cell after 2 days, which was slightly increased to 51.3 fg per cell by day 4, and to 56.5 fg per cell by day 7. Measurements taken over a 7-day period revealed that RA pretreatment with $10^{-10}$ M to $10^{-6}$ M RA did not significantly alter mICAM-1 expression (values ranging from 45.9 to 60.3 fg ICAM-1 per cell on day 2, and 43.6 to 64.9 fg ICAM-1 per cell by day 7).

By day 2, the Hs294T cell line secreted 3.9-fold lower levels of sICAM-1, which did not change significantly following treatment with $10^{-10}$ M to $10^{-6}$ M RA over a 7-day period (values ranging from 10.4 fg per cell to 14.0 fg per cell on day 2, from 16.4 fg per cell to 23.9 fg per cell on day 4, and from 15.5 fg per cell to 22.9 fg per cell by day 7).

### Addition of sICAM-1 to the cytotoxicity assay

Addition of sICAM-1 over a range of concentrations (25–125 ng ml$^{-1}$) to control C8161 cells did not alter their susceptibility, but RA treatment of cells in combination with sICAM-1 addition, significantly reduced lysis in a dose-dependent manner with lysis decreasing from 50.2% to 38% using 75 ng ml$^{-1}$ ($P < 0.05$), and was further reduced to 24.9% ($P < 0.0005$) using the highest concentration of sICAM-1 (125 ng ml$^{-1}$) (Figure 8).

In contrast, addition of sICAM-1 at the 40:1 ratio, using control or RA-treated Hs294T did not alter tumour cell lysis (data not shown).

### DISCUSSION

Previous investigators have reported on the RA-induced increase of mICAM-1 expression in various cell types (Bouillon et al, 1991; Wang et al, 1992; Bassi et al, 1995; Babina et al, 1997), while this study demonstrates a RA-induced increase in ICAM-1 levels in C8161 and Hs294T human melanoma cell lines. Increases in ICAM-1 expression by cytokines (Jackson et al, 1992; Cao et al, 1997; Eisenthal et al, 1998) and ICAM-1 gene transfection (Lefor and Fabian, 1998) have been correlated with an increased susceptibility of tumour cell lysis. Therefore, we have subsequently examined the correlation between ICAM-1

 © 1999 Cancer Research Campaign expression and the susceptibility of RA-treated cell lines to lysis by LAK cells. The data generated from our blocking ICAM-1 antibody studies indicate mICAM-1 to be partially involved in the tumour/PBL interaction of both treated and untreated tumour cells, providing further evidence for the involvement of ICAM-1 in the immune response to tumours (Maio et al, 1989; Vanky et al, 1990; Jackson et al, 1992; Pandolfi et al, 1992). We have demonstrated that this increase in mICAM-1 expression in response to RA treatment cannot be solely responsible for the increase in LAK cell-mediated lysis of RA-treated tumour cells, as addition of blocking ICAM-1 antibody to the cytotoxicity assays does not reduce lysis to the same level of control experiments. It therefore appears that RA modulates the expression of other molecules in addition to ICAM-1 on the tumour cell surface that could ultimately alter the sensitivity of the tumour cells to LAK cell-mediated lysis. Possible candidate molecules which are known to be involved in the tumour/immune response and which may be regulated by RA include ICAM-2, major histocompatibility complex (MHC) class I and II and LFA-3 (Kaufman, 1995; Gwin et al, 1996). However, despite our findings that the tumour cells weakly express MHC class I molecules, we were unable to show that blocking the HLA–ABC complex with antibody over a range of concentrations had any effect on lysis of either cell line (data not shown).

Previous reports indicate mICAM-1 expression in melanoma correlates with an increased risk of metastasis (Johnson et al, 1989; Kageshita et al, 1993). This somewhat conflicting statement may be explained by the existence of soluble ICAM-1 that can interact with LFA-1 on the lymphocyte and block the lymphocyte from interacting with and lysing the tumour cell. Nude mouse models have previously shown melanoma cells to be a source of sICAM-1 (Giavazzi et al, 1992), a finding supported by our ELISA results which show the C8161 and Hs294T cell lines secrete sICAM-1. There is evidence to show that patients with malignant melanoma who have higher levels of sICAM-1 have a reduced survival rate (Harning et al, 1991). It is interesting to note that in our melanoma cell lines, the highly metastatic C8161 cells secrete 4-fold greater levels of sICAM-1 per cell than the poorly metastatic Hs294T cells which may contribute to the greater ability of the C8161 cells to metastasize in the experimental metastasis model (authors' unpublished observations). No reports exist as to the effect of RA on sICAM-1 expression but we have shown levels of secreted ICAM-1 in these two cell lines to be unaffected by RA treatment. One can speculate from such a result that the immunological consequences of RA treatment in these cell lines is not via the induction of sICAM-1 and that the mechanisms of regulation of mICAM-1 and sICAM-1 are independent of one another in these cell lines. This has also been observed by Heymann et al (1995) who reported that the induction of mICAM-1 by certain cytokines had no effect on sICAM-1 expression. The mechanisms involved in the release of sICAM-1 are unclear; however, it is possible that the soluble form arises either by differential RNA splicing, as is true for P-selectin (Ishiwata et al, 1994), but no corresponding mRNA has been observed, or it may arise via enzymatic cleavage of membrane-associated ICAM-1 to form sICAM-1. Proteolytic cleavage appears to be the most likely explanation and has been suggested in an earlier report by Jackson et al (1993) who observed the shedding of sICAM-1 from bladder cancer cells in response to IFN-γ which was accompanied by downregulation of mICAM-1 expression. In a more recent study by Budnik et al (1996) it was shown that the addition of protease inhibitors to keratinocytes had the ability to inhibit production of sICAM-1 supporting a model of proteolytic cleavage in the release of ICAM-1. If proteolytic cleavage is indeed the mechanism for sICAM-1 production, then our data suggests that RA is unable to affect the putative protease(s) required to cleave the membrane form of ICAM-1.

sICAM-1 has been shown to bind to LFA-1 on the LAK cell surface which would ultimately prevent the lymphocytes from interacting with mICAM-1, preventing lysis of the tumour cell. Altomonte et al (1993) reported on the inhibition of melanoma cell lysis by sICAM, whereas Sanchez-Rovira et al (1998) have shown an inhibition of colorectal cancer cell lysis using sICAM-1. However, in contrast to this, we demonstrate that introduction of this soluble form to the lysis assay of both the Hs294T cells and the C8161 cells does not alter lysis, an observation supported by recent data from Young et al (1997), who found that addition of sICAM to the cytotoxicity assays of bladder cancer cell lines had no effect on lysis. Our data indicates sICAM-1 release from these melanoma cells appears to be unimportant in the evasion of the melanoma cells from the immune system in vitro. However, it does not rule out the possibility that in the in vivo situation, sICAM may exist bound to other proteins or as a multimeric form which may have a greater potential to bind to LFA-1 sites on the lymphocyte which could contribute to tumour cell evasion. In contrast to this, sICAM-1 addition to RA-treated C8161 cells reduced lysis, suggesting sICAM-1 interference of the tumour/LAK cell interaction plays a significant role in preventing lysis of RA-treated tumour cells. It appears that the combination of sICAM-1 and the RA-induced alterations in membrane molecules is sufficient to partially prevent lysis of the highly metastatic cells.

In summary, we have demonstrated that RA treatment of C8161 and Hs294T human melanoma cell lines induces these cells to become more susceptible to lysis by LAK cells, and although ICAM-1 is involved in this LAK/tumour cell interaction, the observed RA-induced increase in mICAM-1 expression cannot solely account for the increase in lysis with RA treatment. We have shown sICAM-1 to be secreted by both cell lines, and to be expressed in larger amounts in the highly metastatic cell line compared to the poorly metastatic tumour cells. Addition of sICAM-1 to the assay has no effect on lysis of the tumour cells but reduces lysis of the highly metastatic RA-treated C8161 cells.

Further work will be required to define changes in surface expression of other molecules involved in the LAK/tumour cell response in combination with our current studies analysing the distribution of RA receptors (RAR) in these cell lines to determine whether a relationship exists between RA sensitivity, RAR expression and ICAM-1 expression.

### ACKNOWLEDGEMENT

Claire L Alexander was a recipient of a University of Glasgow Clinical Medical Planning Unit Scholarship.

### REFERENCES

Altomonte M, Gloghini A, Bertola G, Gasparollo A, Carbone A, Ferrone S and Maio M (1993) Differential expression of cell adhesion molecules CD54/CD11a and CD58/CD2 by human melanoma cells and functional role in their interaction with cytotoxic cells. *Cancer Res* **53**: 3343–3348

Aoudjit F, Bousse M, Stratowa C, Voraberger G and Audette M (1994) Regulation of intercellular adhesion molecule-1 expression by retinoic acid: analysis of the 5′ regulatory region of the gene. *Int J Cancer* **58**: 543–549

Babina M, Weber S and Henz BM (1997) Retinoic acids and dexamethasone alter cell-surface density of β-2 integrins and ICAM-1 on human leukemic (HMC-1) mast cells. *Arch Derm Res* **289**: 111–115

Banks RE, Gearing AJH, Hemingway IK, Norfolk DR, Perren TJ and Selby PJ (1993) Circulating ICAM-1, E-selectin and vascular cell adhesion molecule (VCAM) in human malignancies. *Br J Cancer* **68**: 122–124

Bassi V, Vitale M, Feliciello A, Deriu S, Rossi G and Fenzi G (1995) Retinoic acid induces ICAM-1 hyperexpression in human thyroid carcinoma cell lines. *J Clin Endocrinol Metabol* **80**: 1129–1135

Becker JC, Dummer R, Schmidt RE, Burg G and Hartmann AA (1992) Shedding of intercellular adhesion molecule-1 (ICAM-1) from melanoma cell lines – functional consequences on cell-mediated cytotoxicity. *Immun Infect* **20**: 62–63

Bouillon M, Tessier P, Boulianne R, Destrempe R and Audette M (1991) Regulation by retinoic acid of ICAM-1 expression on human tumour cell lines. *Biochim Biophys Acta* **1097**: 95–102

Budnik A, Grewe M, Gyufko K and Krutmann J (1996) Analysis of the production of soluble ICAM-1 molecules by human cells. *Exp Hematol* **24**: 352–359

Cao XT, Chen GY, He L, Zhang WP, Yu YZ and Wang JL (1997) Involvement of MHC class 1 molecule and ICAM-1 in the enhancement of adhesion and cytotoxic susceptibility to immune effector cells transfected with the interleukin (IL)-2, IL-4 or IL-6 gene. *J Cancer Res Clin Oncol* **123**: 602–608

Cilenti L, Toniato E, Ruggiero P, Fusco C, Farina AR, Tiberio A, Hatday AC, Gulino A, Frati L and Martinotti S (1995) Transcriptional modulation of the human intercellular adhesion molecule gene-1 (ICAM-1) by retinoic acid. *Exp Cell Res* **218**: 263–270

Creasey AA, Smith HS, Hackett AJ, Fukuyama K, Epstein WL and Madin SH (1979) Biological properties of human melanoma cells in culture. *In vitro Cell Dev Biol* **15**: 342–350

Eisenthal A, Marder O, Maymon B, Misonzhnik F, Skornick Y, Brazowski E, Czernobilsky B, Walt H and Lifschitz-Mercer B (1998) The effect of interferon gamma and tumour necrosis factor alpha on the expression of ICAM-1 and HLA-DR molecules on cells of a human germ cell neoplasm and their susceptibility to lysis by lymphokine-activated killer cells. *Pathobiology* **66**: 205–208

Gao Z and MacKenzie IC (1996) Influence of retinoic acid on the expression of cytokeratins, vimentin and ICAM-1 in human gingival epithelia in-vitro. *J Peridont Res* **31**: 81–89

Giavazzi R, Chirivi RGS, Garofalo A, Rambaldi A, Hemingway I, Pigott R and Gearing AJH (1992) Soluble intercellular adhesion molecule-1 is released by human melanoma cells and is associated with tumour growth in nude mice. *Cancer Res* **52**: 2628–2630

Gwin JL, Taylor CG, Taylor DD and Eisenberg B (1996) Role of LFA-3, ICAM-1 and MHC class 1 on the sensitivity of human tumour cells to LAK cells. *J Surg Res* **60**: 129–136

Harning R, Mainolfi E, Bystryn JC, Henn M, Merluzzi VJ and Rothlein R (1991) Serum levels of circulating ICAM-1 in human malignant melanoma. *Cancer Res* **51**: 5003–5005

Heymann D, Godard A, Raher S, Ringeard S, Lassort D, Blanchard F and Harb J (1995) Human interleukin for DA cells leukemia inhibitory factor and oncostatin-M enhance membrane expression of intercellular adhesion molecule-1 on melanoma cell but not the shedding of its soluble form. *Cytokine* **7**: 111–117

Ishiwata N, Takio K, Katayama H, Watanabe K, Titani K, Ikeda Y and Handa M (1994) An alternatively spliced isoform of P-selectin is present in vivo as a soluble molecule. *J Biol Chem* **269**: 23708–23715

Jackson AM, Alexandrov AB, Prescott S, James K and Chisholm GD (1992) Role of adhesion molecules in lymphokine-activated killer cell killing of bladder cancer cells: further evidence for a third ligand for leukocyte function associated antigen-1. *Immunology* **76**: 286–291

Jackson AM, Alexandrov AB, Gribben SC, Esuvarnathan K and James K (1993) Expression and shedding of ICAM-1 in bladder cancer cells and its role in immunotherapy. *Int J Cancer* **55**: 921–925

Johnson JP, Stade BG, Holzmann B, Schwable W and Riethmuller G (1989) De novo expression of ICAM-1 in melanoma correlates with increased risk of metastasis. *Proc Natl Acad Sci USA* **86**: 641–644

Kageshita T, Yoshii A, Kimura T, Kuriya N, Ono T, Tsujisaki M, Imai K and Ferrone S (1993) Clinical relevance of ICAM-1 expression in primary lesions and serum of patients with malignant melanoma. *Cancer Res* **53**: 4927–4932

Kaufman DS (1995) Inhibition of selective signalling events in natural killer cells recognising MHC class 1. *Proc Natl Acad Sci (USA)* **92**: 6484–6488

Lefor AT and Fabian DF (1998) Enhanced cytolytic activity of tumour infiltrating lymphocytes (TILs) derived from an ICAM-1 transfected tumour in a murine model. *J Surg Res* **75**: 49–53

Maio M, Tessitori G, Pinto A, Temponi M, Colombatti A and Ferrone S (1989) Differential role of distinct determinants of ICAM-1 in immunologic phenomena. *J Immunol* **143**: 181–188

Marlin SD and Springer TA (1987) Purified intercellular adhesion molecule-1 (ICAM-1) is a ligand for lymphocyte function associated antigen (LFA-1). *Cell* **51**: 813–819

Pandolfi F, Trentin L, Boyle LA, Stamenkovie I, Byres HR, Colvin RB and Kurnick TJ (1992) Expression of cell adhesion molecules in human melanoma cell lines and their role in cytotoxicity mediated by tumour infiltrating lymphocytes. *Cancer* **69**: 1165–1173

Rothlein RM, Czajkowski MM, O'Neili SD, Marlin E, Mainolfi EA and Merluzzi VJ (1988) Induction of intercellular adhesion molecule-1 on primary and continuous cell lines by pro-inflammatory cytokines. Regulation by pharmacologic agents and neutralizing antibodies. *J Immunol* **141**: 1665–1669

Rothlein RM, Mainolfi EA, Czajkowski M and Marlin SD (1991) A form of circulating ICAM-1 in human serum. *J Immunol* **147**: 3788–3793

Sanchez-Rovira P, Jimenez E, Carracedo J, Barneto IC, Ramirez R and Aranda E (1998) Serum levels of intercellular adhesion molecule 1 (ICAM-1) in patients with colorectal cancer: inhibitory effect on cytotoxicity. *Eur J Cancer* **34**: 394–398

Smith MA, Parkinson DR, Cheson BD and Friedman MA (1992) Retinoids in cancer therapy. *J Clin Oncol* **10**: 839–864

Staunton DE, Marlin SD, Stratowa C, Dustin ML and Springer TA (1988) Primary structure of ICAM-1 demonstrates interaction between members of the immunoglobulin and integrin supergene families. *Cell* **52**: 925–933

Vanky F, Wang P, Patarroyo M and Klein E (1990) Expression of adhesion molecule ICAM-1 and MHC class 1 antigens on human tumour cells is required for their interaction with autologous lymphocytes in vitro. *Cancer Immunol Immunother* **31**: 19–27

Wang ZG, Cao Y, Durso CM and Ferrone S (1992) Differential susceptibility of cultured human melanoma cell lines to enhancement by retinoic acid of intercellular adhesion molecule-1 expression. *Cancer Res* **52**: 4766–4772

Webb DSA, Mostowski HS and Gerrard TL (1991) Cytokine induced enhancement of ICAM-1 expression results in increased vulnerability of tumour cells to monocyte mediated lysis. *J Immunol* **146**: 3682–3686

Welch DR, Bisi JE, Miller BE, Conaway D, Seftor EA and Yohem KH (1991) Characterisation of a highly invasive and spontaneously metastatic human malignant melanoma cell line. *Int J Cancer* **47**: 227–237

Young DG, Jackson AM and James K (1997) Purification and characterisation of soluble intercellular adhesion molecule-1 (sICAM-1) and its effect on cell mediated cytolysis of tumour cells. *Int J Oncol* **10**: 827–834

*© 1999 Cancer Research Campaign*
